# Analytical Technologies for Milk Component Analysis: A Review of Progress and Challenges

**DOI:** 10.3390/foods15152752

**Published:** 2026-08-05

**Authors:** Ying Zhang, Qiang Jiang, Wenjun Zhao, Yujie Wang, Xiaochao Wei, Yali Zhang, Xiuge Wang, Jinming Huang

**Affiliations:** 1 Institute of Animal Science and Veterinary Medicine, Shandong Academy of Agricultural Sciences, Jinan 250100, China15628995256@163.com (Y.W.);; 2 College of Food Science and Nutritional Engineering, China Agricultural University, Beijing 100193, China

**Keywords:** dairy products, milk component detection, rapid detection, multi-omics, quality and safety

## Abstract

Dairy products are an important source of high-quality protein, calcium, and various micronutrients in the human diet. The detection of milk components spans the entire industrial chain, including raw milk quality control, processing management, and market supervision. It has evolved from traditional pass/fail judgment toward an integrated approach encompassing risk early warning, quality enhancement, and traceability and authentication. This paper reviews the classification of dairy components targeted for detection and summarizes major technical systems, including conventional physicochemical methods, chromatography and spectroscopy techniques, biosensing and microfluidic rapid detection, as well as multi-omics-based deep profiling. The principles, advantages, and application limitations of each method are compared. Key challenges such as complex matrix interference, lack of reference standards, and standardization of rapid detection methods are analyzed. Future directions toward intelligent, on-site, precise, and panoramic detection are also discussed, aiming to provide technical references for quality and safety control, quality improvement, and high-quality development of the dairy industry.

## 1. Introduction

As an important source of high-quality protein, calcium, and various micronutrients in the global dietary structure, dairy products play an irreplaceable role in ensuring human nutrition and health [1]. In recent years, driven jointly by consumption upgrading, technological innovation, and the concept of sustainable development, the global dairy industry has been undergoing profound transformation, while simultaneously facing multiple challenges such as supply–demand fluctuations, safety risks, and structural contradictions [2].

Developed countries have established comprehensive legal frameworks for dairy safety and full-chain regulatory mechanisms. The European Union implements the “from farm to fork” end-to-end control system [3], while the United States mandates the Hazard Analysis and Critical Control Point (HACCP) system [4]. Current dairy safety risks exhibit regional differentiation: developed countries focus on adulteration traceability and emerging environmental contaminants as prevention and control priorities [5]; in contrast, developing countries continue to face prominent issues such as pathogenic bacterial contamination, antibiotic residues, excessive aflatoxin M1 levels, and traditional adulteration practices [6].

China is the world’s third-largest milk producer and the largest consumer market for dairy products. The qualified rate of raw milk in spot checks has remained above 99.9% for many consecutive years, with overall quality and safety indicators reaching internationally advanced levels. Since 2008, China has achieved a leapfrog improvement in dairy quality and safety by refining laws and regulations, implementing the dairy industry revitalization plan, and enforcing full-chain supervision [7].

Over the past decade, several reviews have addressed various aspects of milk component analysis, yet a comprehensive and systematically structured overview that bridges classical methods, emerging technologies, and multi-omics platforms remains lacking. Existing reviews have largely focused on specific technological domains: Acquavia et al. [8] provided a comprehensive summary of extraction and analytical methods for milk components, with an emphasis on sample preparation techniques; Loudiyi et al. [9] reviewed spectroscopic methods (MIR, NIR, Raman, fluorescence) combined with chemometric modeling for predicting the functional properties of raw bovine milk; van Asselt et al. [10] examined food safety hazards across the European dairy supply chain from a regulatory perspective; and Wijayanti et al. [11] summarized enzyme-based biosensors for lactose detection in milk samples. However, several critical gaps persist in the current literature. First, no single review has systematically compared classical physicochemical methods, chromatographic techniques, spectroscopic platforms, biosensing technologies, and multi-omics approaches within a unified analytical framework, making it difficult for researchers and practitioners to select appropriate methods for specific applications [12]. Second, the rapid advancement of microfluidic digital PCR, ion mobility-mass spectrometry, and metagenomic sequencing in the past 5 years has outpaced the coverage of existing reviews, leaving a significant knowledge gap regarding the practical utility and limitations of these emerging technologies for routine dairy analysis [13]. Third, the integration of multi-omics data with traditional quality parameters and health monitoring indicators has not been critically evaluated in a dairy-specific context, despite growing interest in precision livestock farming and personalized nutrition [14]. Fourth, the systemic challenges of method translation—including economic viability, workforce capacity, data interoperability, and regulatory adaptation—have received insufficient attention in previous reviews, which tend to focus on technical performance metrics rather than practical implementation barriers [15]. Fifth, the increasing demand for on-site, rapid, and multi-analyte detection in decentralized dairy supply chains (particularly in developing countries) necessitates a critical assessment of the trade-offs between analytical sophistication and practical utility—a dimension that remains underexplored in the existing literature [16]. Collectively, these gaps underscore the need for a comprehensive, systematically structured review that not only surveys available analytical technologies but also provides a decision-making framework for method selection and critically evaluates the translational challenges that constrain the adoption of advanced technologies in routine dairy quality and safety control. The present review aims to address these gaps by providing a structured analysis of detection technologies across three orthogonal dimensions—application scenario, technology type, and detection target—while critically examining the economic, regulatory, and workforce challenges that shape the practical implementation landscape.

The economic impact of dairy adulteration and contamination is substantial. According to the Food and Agriculture Organization (FAO), food fraud—including dairy adulteration—costs the global food industry an estimated $10–$15 billion annually [17]. In the dairy sector alone, the 2008 melamine contamination incident in China caused direct economic losses exceeding RMB 20 billion (approximately $3 billion), with long-term damage to consumer confidence and export markets estimated to be far higher. Antibiotic residues in milk, resulting from improper veterinary drug use, not only pose direct health risks but also lead to significant economic losses through milk rejection, processing disruptions, and trade restrictions. The economic burden of mastitis, a major driver for milk component testing (particularly somatic cell count), is estimated at $35–50 billion globally per year, encompassing reduced milk yield, increased culling rates, treatment costs, and decreased milk quality premiums [18].

Milk component detection covers the entire industrial chain: in upstream dairy farms, it is used for raw milk grading and pricing, precision feeding, and dairy cow health monitoring; in midstream processing, it is applied to raw material acceptance, process control, and product standardization; in downstream markets, it serves compliance assessment, authenticity identification, and consumer trust building [19].

From an economic perspective, analytical testing for milk components serves multiple cost-benefit functions: (1) Risk mitigation—early detection of contaminants prevents large-scale recalls, liability claims, and brand damage; (2) Quality-based pricing—component analysis enables premium payments for high-quality milk, incentivizing improved farm management; (3) Process optimization—real-time monitoring reduces waste and enhances processing efficiency; (4) Export market access—compliance with international standards through validated analytical methods is a prerequisite for the dairy trade, with developing countries often facing annual export losses of $100–500 million due to non-compliance with maximum residue limits (MRLs) and other quality specifications.

As a complex biological fluid, bovine milk constituents can be categorized into three major groups according to analytical targets: macronutrients, micronutrients together with functional components, and hazardous risk factors, which comprehensively satisfy the requirements for nutritional quality evaluation, functional property characterization, and food safety regulation.

Macronutrients account for 10–13% of the total mass of milk and serve as core indicators for quality evaluation, processing suitability, and nutritional labeling. In fermented dairy product processing, genetic polymorphism influences digestibility and absorption, and whey protein exhibits favorable functional properties for processing [20]. Lactose is the predominant carbohydrate in milk, accounting for 4.2–5.0% of total mass, and represents an important energy source in milk. Driven by the prevalence of lactose intolerance in the population, low-lactose and lactose-free dairy products are undergoing rapid development [21].

Micronutrients and functional components are characterized by low concentrations, diverse structures, and large variations in chemical properties, which impose high demands on sample pretreatment and detection techniques [22]. The Table 1 presents the detection strategies for these components:

Risk factors are classified into two broad categories: endogenous risk factors (derived from cow physiology and pathology) and exogenous risk factors (originating from environment, farming operations, and intentional adulteration), both of which directly affect dairy safety, processing suitability, and shelf-life. Endogenous factors include: (1) Somatic cell count (SCC)—a core indicator for diagnosing udder health; elevated SCC levels (>200,000 cells/mL) indicate intramammary infection, reduce milk component contents, impair processing characteristics (e.g., decreased cheese yield, prolonged rennet coagulation time), and shorten product shelf-life. (2) Pathological metabolites—such as BHBA and acetone, which accumulate in milk during ketosis, a common metabolic disorder in high-yielding dairy cows, affecting milk flavor and processing properties [28]. Exogenous factors include: (1) Antibiotic residues—caused by improper medication use or insufficient withdrawal periods, representing a strictly controlled safety indicator at the farming stage; β-lactams, tetracyclines, and sulfonamides are the most commonly detected residues [29]. (2) Microbial contamination—pathogenic bacteria (e.g., *Salmonella* spp., *Escherichia coli O157:H7*, *Listeria monocytogenes*) can cause foodborne illnesses, while heat-resistant enzymes (proteases and lipases) produced by psychrophilic bacteria (e.g., *Pseudomonas* spp.) lead to age gelation, flavor deterioration, and quality loss during refrigerated storage and shelf-life [30]. (3) Chemical contaminants—including aflatoxin M1 (a metabolite of aflatoxin B1 from contaminated feed), heavy metals (lead, arsenic, mercury), and pesticide residues, which are subject to stringent regulatory limits. (4) Adulterants—intentional addition of cheaper substances (e.g., melamine, vegetable oils, starch, urea, and foreign proteins) for economic gain, which poses serious food safety risks, as exemplified by the 2008 melamine crisis.

## 2. Literature Search Strategy

A comprehensive literature search was performed to identify relevant original research articles, review papers, and established analytical protocols for milk component detection. The search was conducted using three electronic databases: Web of Science Core Collection, PubMed, and China National Knowledge Infrastructure (CNKI). The selection of these databases ensured comprehensive coverage of both international peer-reviewed literature—with Web of Science providing broad multidisciplinary coverage, and PubMed offering in-depth biomedical and food science indexing—and regionally significant publications from China, a major dairy-producing country. The retrieval timespan was set from the earliest available records in each database to June 2026, with specific coverage periods as follows: Web of Science (1990–June 2026), PubMed (1990–June 2026), and CNKI (1980–June 2026).

### 2.1. Search Strategy and Keywords

Search keywords were developed to capture the full scope of analytical technologies applied to milk component analysis. The primary search terms combined three conceptual groups using Boolean operators:

Component terms: “milk component” OR “dairy component” OR “milk composition” OR “milk constituent” OR “milk protein” OR “milk fat” OR “lactose” OR “oligosaccharide” OR “vitamin” OR “mineral” OR “antibiotic residue” OR “mycotoxin” OR “pathogen” OR “somatic cell count” OR “adulterant*”

Technology terms: “analytical method” OR “detection method” OR “chromatograph” OR “HPLC” OR “GC” OR “HPAEC-PAD” OR “mass spectrometr” OR “MS” OR “spectroscop” OR “FTIR” OR “NIR” OR “MIR” OR “biosensor” OR “immunosensor” OR “microfluidic” OR “digital PCR” OR “metabolomics” OR “proteomics” OR “lipidomics” OR “metagenomics” OR “multi-omics”

Matrix terms: “milk” OR “dairy” OR “raw milk” OR “milk powder” OR “fermented milk” OR “yogurt” OR “cheese”

These keyword groups were combined as: (Component terms) AND (Technology terms) AND (Matrix terms). Additional targeted searches were performed for specific emerging technologies using terms including “ion mobility spectrometry”, “UHPLC-IMS-HRMS”, “droplet digital PCR”, “CRISPR-based detection”, and “surface-enhanced Raman scattering”. For PubMed-specific searches, MeSH (Medical Subject Headings) terms were incorporated where applicable, including “Milk/chemistry”, “Food Analysis/methods”, “Chromatography, High Pressure Liquid”, “Mass Spectrometry”, and “Biosensing Techniques”, to optimize retrieval precision.

### 2.2. Eligibility Criteria

Studies were included in this review if they met the following criteria. Included were original experimental articles or comprehensive reviews focusing on qualitative or quantitative detection of milk components—including macronutrients, functional small molecules, lipids, proteins, and contaminants—in dairy matrices such as raw milk, milk powder, and fermented dairy products. Only full-text papers published in English or Chinese from the aforementioned electronic databases were considered. Eligible studies were required to report complete analytical performance indicators, including detection limit, recovery, precision, and pretreatment parameters, to enable cross-comparison of detection technologies. Additionally, studies must have been published in peer-reviewed journals or as official standard methods.

Conversely, studies were excluded if they fell into any of the following categories: narrative reviews, patents, conference abstracts, or theses lacking complete original experimental data; research objects unrelated to dairy milk component detection (e.g., cell culture models, human plasma, plant-based beverages); articles missing core experimental parameters (such as detection limit or recovery data) that precluded horizontal technical comparison; duplicate publications based on identical experimental datasets; and studies focused exclusively on non-analytical aspects, such as animal nutrition or dairy processing without method performance data.

### 2.3. Screening Process

The literature screening process was conducted in three stages. First, all search records were exported to reference management software, and duplicates were removed automatically. Second, titles and abstracts were screened manually against the eligibility criteria. Articles that were clearly irrelevant (e.g., studies on non-dairy matrices, clinical applications, or animal physiology without analytical method validation) were excluded at this stage. Third, the full texts of potentially eligible articles were retrieved and assessed in detail. For each full-text article, the following information was extracted: detection target, analytical technique, sample matrix, pretreatment method, detection limit (LOD), limit of quantification (LOQ), recovery range, precision (RSD/RSDr), and key method characteristics. Data extraction was performed by two independent reviewers, with discrepancies resolved through discussion and consensus.

### 2.4. Study Selection and Characteristics of Included Studies

After deduplication and initial screening, eligible original studies were identified and supplemented with relevant reviews, standard methods, and regulatory documents to form the final reference corpus. A total of 124 references are cited in this review, with original experimental studies constituting the main body, complemented by foundational methodological papers, authoritative reviews, and regulatory documents to provide comprehensive technical comparison and theoretical background.

The cited literature covers a diverse range of dairy matrices, detection targets, and analytical techniques. Regarding matrices, the studies encompassed raw bovine milk, pasteurized milk, ultra-high temperature (UHT) treated milk, milk powder, infant formula, and fermented dairy products such as yogurt and cheese. Regarding detection targets, the literature addressed: (1) macronutrients, including proteins (total protein, casein, whey protein), fats (total fat, fatty acid profiles, triglycerides), and lactose; (2) micronutrients and functional components, including vitamins (water-soluble and fat-soluble), minerals and trace elements, oligosaccharides, and enzyme activities; (3) hazardous risk factors, including antibiotic residues (β-lactams, tetracyclines, sulfonamides), mycotoxins (aflatoxin M1), pathogenic bacteria (*Salmonella* spp., *Escherichia coli O157:H7*, *Listeria monocytogenes*, *Staphylococcus aureus*), and adulterants (melamine, urea, foreign proteins).

Regarding analytical techniques, the cited literature spans four major methodological families: (1) chromatographic methods (HPLC-UV/DAD, GC-FID, HPAEC-PAD, UHPLC-MS/MS, HILIC-MS); (2) spectroscopic techniques (FTIR, NIR, Raman, fluorescence spectroscopy); (3) biosensing platforms (enzyme-based biosensors, electrochemical immunosensors, optical immunosensors); and (4) microfluidic, digital PCR, and multi-omics approaches (metabolomics, proteomics, metagenomics). Geographically, the studies originated primarily from Europe, North America and Asia, reflecting the concentration of dairy research in major milk-producing regions.

A summary of key characteristics of the cited literature—including detection target, analytical technique, matrix, and key performance parameters—is provided in Supplementary Table S1 to facilitate cross-comparison and to enable readers to assess the evidence base underlying the technical comparisons presented in subsequent sections.

### 2.5. Quality Assessment and Reporting

Given the focus on analytical method performance rather than clinical or intervention outcomes, the quality of included studies was assessed based on the completeness of reported analytical parameters. Studies were considered methodologically robust if they reported at least the following parameters: detection limit (LOD) or limit of quantification (LOQ), recovery rate (expressed as percentage recovery of spiked analytes), precision (relative standard deviation, RSD, or standard deviation), and sufficient description of sample pretreatment and chromatographic/separation conditions. Studies that reported partial parameters but were considered pioneering or methodologically influential were retained for qualitative discussion with appropriate caveats.

The reporting of this literature search strategy follows the principles of systematic review methodology adapted for technology-focused reviews in analytical chemistry. The search strategy, eligibility criteria, screening process, and study characteristics are described in sufficient detail to enable replication. The completed search and screening records are available from the corresponding author upon reasonable request.

This narrative review has inherent limitations that should be acknowledged. Unlike systematic reviews employing exhaustive, protocol-driven search strategies and quantitative synthesis, narrative reviews are subject to potential selection bias in study identification and inclusion. While we have endeavored to ensure broad and representative coverage of major analytical technologies for milk component analysis—including classical physicochemical methods, chromatography, spectroscopy, biosensing, microfluidics, and multi-omics—the selection of studies inevitably reflects the authors’ expertise, judgment, and thematic focus. This review should therefore be viewed as a structured, critical overview providing a decision-making framework and balanced assessment of available technologies, rather than an exhaustive inventory of all published studies. Readers are encouraged to consult the cited primary literature and systematic reviews for comprehensive coverage of specific methodological domains. We have emphasized emerging technologies and recent advances (2020–2026) to complement established methods, aiming to present a forward-looking perspective on the field.

## 3. Traditional Classical Detection Methods

Traditional detection methods serve as the foundation and reference standards for milk component analysis. They mainly encompass three major technical directions: basic physicochemical detection, chromatographic separation analysis, and infrared spectroscopy rapid detection. These methods are characterized by high maturity, good accuracy, and a high degree of standardization.

### 3.1. Basic Physicochemical Detection Methods

Protein content is one of the core indicators for quality evaluation of dairy products. The internationally recognized reference methods for milk protein determination are the Kjeldahl method and the Dumas combustion method, both of which are based on total nitrogen determination followed by conversion to protein content using a conversion factor (specific milk protein factor of 6.38). The Kjeldahl method involves digestion of the sample by heating with concentrated sulfuric acid, converting organic nitrogen into ammonium sulfate, followed by alkalinization, distillation, and quantification of the released ammonia. The Dumas method, in contrast, achieves complete combustion of the sample under high-temperature, oxygen-rich conditions, with nitrogen quantitatively determined using a thermal conductivity detector.

**Figure 1:**
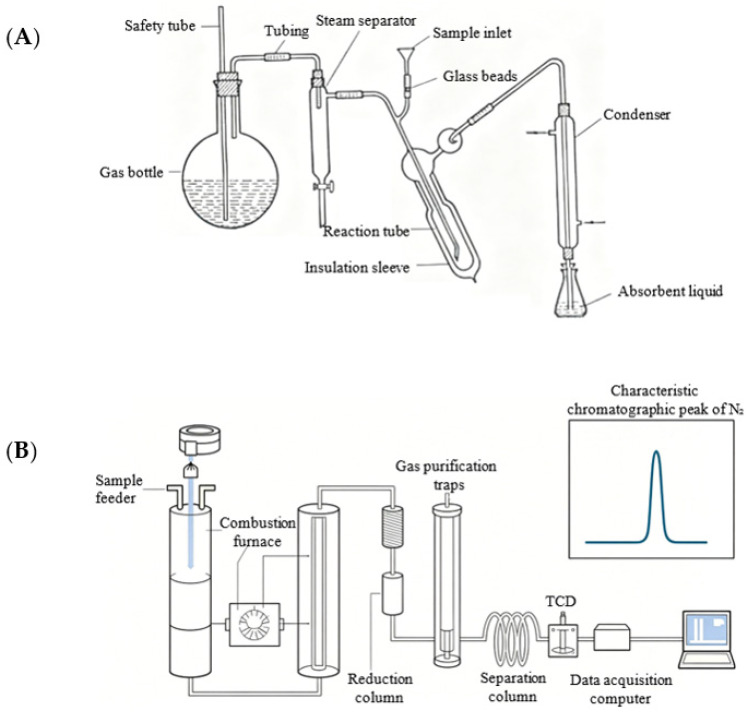
Workflow of Kjeldahl method and Dumas method. (**A**) Parnas–Wagner micro-Kjeldahl steam distillation apparatus for the classic Kjeldahl nitrogen determination method, redrawn with reference to the standard experimental diagram described in Settle F A. Handbook of Analytical Chemistry Laboratory Practice [M]. New York: Academic Press, 2018 [33]. (**B**) Dumas combustion elemental analysis system for rapid total nitrogen measurement. The structural principle refers to publicly available standard instrument diagrams on the internet, and all lines, labels and color schemes have been manually redrawn and revised to meet journal graphic specifications.

In addition to the Soxhlet and Röse–Gottlieb methods, the Gerber method (also known as the butyrometric method) is widely used in the dairy industry for routine fat determination in liquid milk samples. The Gerber method employs sulfuric acid (density 1.82–1.84 g/mL) to digest proteins and dissolve lactose, followed by the addition of isoamyl alcohol to facilitate fat separation. After centrifugation in a butyrometer, the fat content is directly read from the graduated scale. The Gerber method offers several practical advantages for liquid milk analysis: (1) rapid analysis time (approximately 5–10 min per sample), (2) no requirement for drying or pre-extraction of the sample—directly applicable to liquid milk, (3) simple equipment and low cost, and (4) suitable for routine quality control in processing plants. However, the method has notable limitations: (1) accuracy is lower than gravimetric reference methods—the fat volume read includes non-fat substances and is affected by temperature and operator technique, (2) use of concentrated sulfuric acid poses significant safety hazards, (3) it is not suitable for low-fat or fermented dairy products due to sample-specific correction factors, and (4) results show inter-operator variability. Despite these limitations, the Gerber method remains the most common routine fat test in the global dairy industry, particularly in liquid milk processing plants, due to its speed and simplicity. A comparison of the advantages and disadvantages of these three methods is presented as Table 2. The workflow of the Kjeldahl method and the Dumas method is showed in Figure 1.

The Kjeldahl and Dumas methods measure total nitrogen, failing to distinguish protein nitrogen from non-protein nitrogen (NPN)—a flaw exploited in adulteration. Urea (46.6% N) and melamine (66.6% N) are water-soluble, nitrogen-rich compounds that inflate apparent protein content when added to milk, with 0.1% urea raising protein estimates by ~0.3 percentage points, and melamine being even more potent. The 2008 Chinese melamine crisis, affecting ~300,000 infants with six deaths, highlighted this risk. Confirmatory tests such as dye-binding, amino acid analysis, or LC-MS/MS are now used to differentiate authentic protein from NPN, closing the loophole for regulatory enforcement.

The Soxhlet extraction method is based on the principle of solid-liquid extraction and is suitable for the determination of free fat in dried or semi-dried dairy products. The Röse–Gottlieb method (alkaline ether extraction method) is applicable to a variety of dairy products, including liquid milk, milk powder, condensed milk, and fermented milk. The Table 3 presents three methods.

### 3.2. Chromatographic Detection Techniques

High-performance liquid chromatography (HPLC) and gas chromatography (GC) are the most widely applied chromatographic separation techniques in milk component analysis. HPLC is suitable for the analysis of non-volatile, thermally unstable, or strongly polar milk components, and serves as the mainstream technique for the quantification of nutritional and functional components in milk, including vitamins, oligosaccharides, nucleotides, amino acids, and proteins [8].

Carbohydrate compounds lack ultraviolet-absorbing chromophores and exhibit significant differences in polarity, making them difficult to separate and detect directly by conventional reversed-phase chromatography. High-performance anion-exchange chromatography with pulsed amperometric detection (HPAEC-PAD) and ultra-high-performance liquid chromatography-mass spectrometry (UHPLC-MS) have become the two pillar technologies for carbohydrate analysis, owing to their advantages of no derivatization requirement, high sensitivity, and isomer resolution capability.

HPAEC-PAD is based on the property that carbohydrates exist as anions under strongly alkaline conditions, achieving separation by anion-exchange columns and quantification using a pulsed amperometric detector. Its core advantages include no need for derivatization, high sensitivity, and excellent separation of structurally similar carbohydrate isomers; however, it cannot provide molecular mass information.

**Figure 2:**
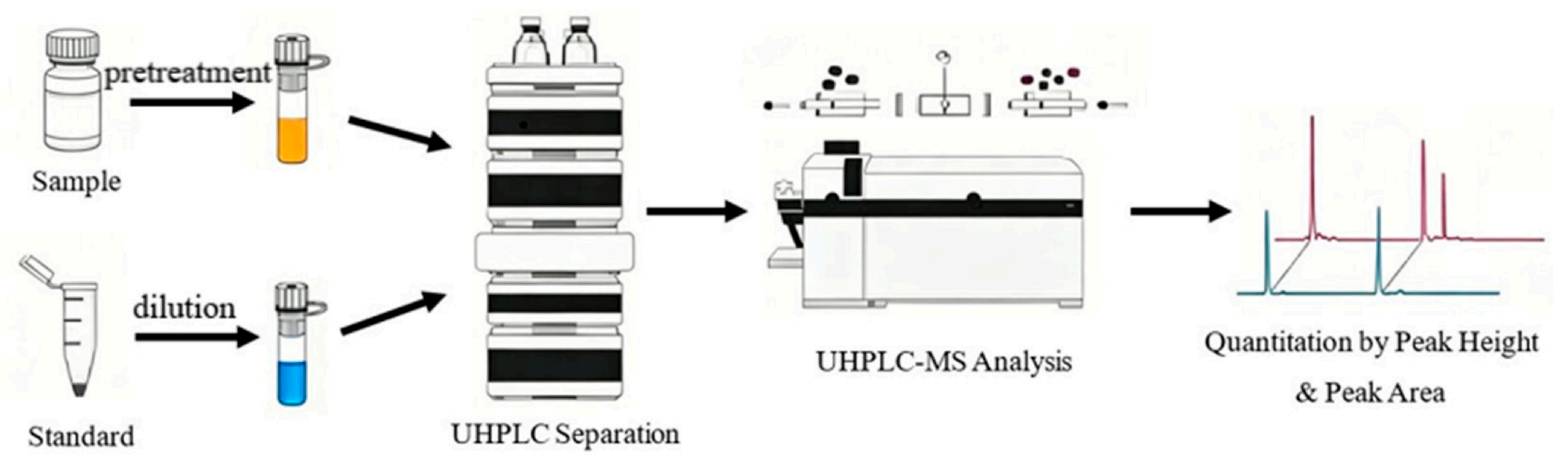
Workflow of UHPLC-MS analysis.

UHPLC-MS compensates for the limitations of HPAEC-PAD by coupling hydrophilic interaction liquid chromatography (HILIC) or reversed-phase chromatography with high-resolution mass spectrometry. Using UPLC-MS/MS, isomer-level resolution and quantification of 21 natural human milk oligosaccharides can be achieved within 12 min, with detection limits reaching the ng/L level [36]. A comparison of the two technologies is presented as Table 4 and Figure 2 shows Workflow of UHPLC-MS analysis.

Hydrophilic interaction liquid chromatography (HILIC) is a liquid chromatographic mode specifically designed for the separation of strongly polar and hydrophilic compounds. Its core mechanism involves the use of a polar stationary phase in conjunction with a mobile phase system consisting of a high proportion of organic solvent and water, forming a dynamic “water-enriched layer” on the stationary phase surface. The target analytes are retained through partitioning between this water-enriched layer and the mobile phase [38]. HILIC exhibits excellent orthogonal complementarity to reversed-phase liquid chromatography (RPLC) and demonstrates irreplaceable technical advantages in the analysis of polar components in dairy products, such as oligosaccharides, nucleotides, and polar metabolites [39].

Nucleosides and nucleotides are common functional ingredients added to infant formula powders, as well as naturally occurring bioactive substances in human milk. However, these compounds are highly polar and exhibit very weak retention in RPLC. Moreover, phosphorylated nucleotides are prone to adsorption in chromatographic systems, leading to reduced detection sensitivity and reproducibility [40].

### 3.3. Infrared Spectroscopy for Rapid Detection

Mid-infrared (MIR) spectroscopy and near-infrared (NIR) spectroscopy are the most widely applied optical analysis techniques in the field of rapid milk component detection. Both are based on the principles of molecular vibrational spectroscopy, achieving simultaneous quantitative analysis of multiple components such as fat, protein, and lactose by measuring the absorption characteristics of organic molecules in milk samples at specific infrared wavelengths. Table 5 shows the two techniques each offer distinct advantages in milk component analysis and exhibit a complementary relationship:

In practical applications within the dairy industry, MIRS is the mainstream technology and is widely adopted by dairy processing enterprises and Dairy Herd Improvement (DHI) systems worldwide [45,46]. NIRS, on the other hand, holds unique value in the non-destructive testing of solid samples such as milk powder and in adulteration identification using hyperspectral imaging [47].

DHI system provides a decision-making basis for breeding, feeding, and health management on dairy farms through regular measurement of individual cow milk yield, milk components, and somatic cell count. Fourier-transform mid-infrared spectroscopy (FTIR), leveraging its technical advantages of speed, batch processing capability, low cost, and high throughput, has become the core analytical technology within the DHI system.

In DHI laboratories, FTIR milk component analyzers can complete the analysis of a single sample within seconds, simultaneously generating over 30 parameters, including milk fat, protein, lactose, urea nitrogen, and somatic cell count. Among these, milk urea nitrogen can be used to evaluate dietary protein utilization efficiency, while somatic cell count combined with spectral information can assist in subclinical mastitis screening [48].

Differences in instrument models and service years across DHI laboratories can cause systematic spectral deviations, prompting the development of spectral standardization methods. Lactose, the predominant milk carbohydrate (4.2–5.0%), is not only a nutritional marker but also a sensitive biomarker for udder health. Mechanistically, lactose is synthesized exclusively in mammary epithelial cells by lactose synthase. During intramammary infection, inflammatory cytokines (TNF-α, IL-1β, IL-6) compromise the milk–blood barrier and tight junction integrity, impairing lactose synthase activity. Consequently, milk lactose drops from 4.5–5.0% to below 4.2% in subclinical mastitis, often preceding somatic cell count (SCC) elevations by 1–3 days. This early-warning capability makes FTIR-based lactose monitoring in DHI programs a cost-effective screening tool for subclinical mastitis, especially valuable in large herds where individual SCC testing is impractical.

Despite the widespread adoption of FTIR spectroscopy in DHI systems and dairy processing plants, several practical aspects of its implementation warrant critical examination. Key validation metrics for FTIR-based milk analysis include: (1) accuracy—assessed against reference methods (e.g., Kjeldahl for protein, Röse–Gottlieb for fat), typically requiring bias within ±2% for major components; (2) precision—repeatability (RSD < 1%) and reproducibility (RSD < 2%); (3) detection limits—fat and protein at <0.1% *w*/*w*, while trace constituents (acetone, BHBA) require optimized regions with higher LODs (0.02–0.05 mmol/L); and (4) linearity—calibration ranges must cover expected concentrations in commercial samples.

Calibration models, typically developed using partial least squares (PLS) regression with large reference datasets (>1000 samples), face several challenges: (1) matrix effects—fat globule size, temperature, and homogenization affect spectra, requiring standardized sample handling; (2) geographic/seasonal variability—models may not transfer across regions or seasons; (3) instrument-to-instrument transfer—spectral differences require transfer algorithms or re-calibration; and (4) temporal drift—aging, lamp degradation, and cuvette contamination necessitate regular model updating.

Standardization efforts protocols for FTIR milk analysis and the MilkSENSE network for cross-laboratory comparability. Current limitations persist: (1) lower accuracy for trace components (fatty acids, lactoferrin) versus major components; (2) suboptimal prediction accuracy for individual unsaturated/long-chain fatty acids; (3) inability to distinguish protein nitrogen from non-protein nitrogen; and (4) high instrument costs ($60,000–150,000) limit accessibility for small-scale operations. Future developments in portable FTIR, model-sharing platforms, and machine learning-enhanced interpretation are expected to further expand its utility.

The Table 6 summarizes the impact of FTIR-determined milk components on health monitoring.

## 4. Novel Rapid Detection Technologies

Novel rapid detection technologies are characterized by high specificity, short assay time, and portability, making them suitable for scenarios such as on-farm screening, raw milk acceptance testing, and market emergency detection [57]. They mainly encompass two major technical directions: biosensing and microfluidic chips [13].

Sample pretreatment is essential before biosensor detection due to raw milk’s complex matrix (fat 3–5%, protein 3–4%, lactose 4–5%, somatic cells, bacteria), which causes interference via non-specific protein adsorption, fat-induced optical scattering, and endogenous redox-active compounds [58]. Selection depends on biosensor type, target, and application. Common strategies include: (1) Skimming/defatting—centrifugation (3000–10,000× *g*, 10–15 min) or ultrafiltration; essential for optical sensors and microfluidics. (2) Deproteinization—TCA/acetonitrile precipitation or enzymatic digestion (proteinase K); required for electrochemical small-molecule detection to prevent electrode fouling. (3) Dilution (1:5 to 1:100)—simple matrix reduction; suitable for high-concentration targets (lactose, urea). (4) Filtration (0.22–0.45 μm)—removes cells/particles; critical for microfluidic systems. (5) Immunomagnetic separation (IMS)—antibody-functionalized beads for pathogen/allergen enrichment and matrix removal. (6) Solid-phase extraction (SPE)—C18 or HLB cartridges for antibiotics/mycotoxins. (7) Enzymatic pretreatment—lactase/protease hydrolysis or β-galactosidase for glucose-based detection. Selection balances removal efficiency (minimize target loss), simplicity (field use), compatibility, and cost. On-site applications favor dilution/filtration; lab-based use employs IMS/SPE for higher sensitivity and specificity.

### 4.1. Biosensing Detection Technology

Enzyme-based biosensors combine enzymes as biological recognition elements with physical transducers (e.g., electrochemical, optical), and have made significant progress in lactose quantification and antibiotic residue screening due to their high specificity, fast response, and ease of operation. Lactose is not only a basic indicator for nutritional evaluation but also a biomarker of subclinical mastitis. A comparison of various enzyme-based biosensors applied to lactose detection is presented as Table 7.

Excessive antibiotic residues can trigger allergic reactions and promote the dissemination of bacterial resistance. Representative enzyme biosensing technologies in this field include: a nanozyme sensor based on the inhibition by quinolones of Nd(III)-phenylboronic acid nanozyme phosphatase activity (receptor-free/label-free, strong anti-interference capability), and a spore germination sensor based on the chromogenic reaction of β-lactamase produced upon spore germination (capable of simultaneous detection of 12 β-lactam antibiotics with detection limits of 1–1000 ppb).

Immunosensors combine antigen–antibody specific recognition with physical transduction technologies, demonstrating advantages of high specificity, high sensitivity, and rapid response in the fields of milk source protein adulteration identification and pathogenic microorganism detection [16]. The detection of milk source protein adulteration primarily targets the practice of adulterating expensive dairy products such as goat milk and donkey milk with cheaper cow milk. The detection targets—bovine IgG and bovine κ-casein—were selected based on the following criteria: (1) Bovine IgG is the most abundant immunoglobulin in bovine milk and serves as a reliable species-specific marker due to significant structural differences from IgG of other mammals (caprine, ovine, equine, etc.) [19]. (2) Bovine κ-casein is encoded by the CSN3 gene, which exhibits species-specific polymorphisms allowing differentiation between bovine and non-bovine milk proteins [62]. (3) Both targets remain stable under typical milk processing conditions and are not significantly affected by heat treatment, making them robust markers for adulteration detection in pasteurized, UHT, and powdered milk products [63]. (4) These markers are present at relatively high concentrations (IgG: 0.3–1.0 mg/mL; κ-casein: ~1.5–2.5 g/L), enabling convenient detection without extensive sample enrichment. The economic motivation for adulteration testing is substantial: goat milk and donkey milk command 3–5 times higher market prices than bovine milk in many markets, creating a significant incentive for economically motivated adulteration (EMA) with cheaper bovine milk [64]. A comparison of the performance of representative immunosensors is presented as Table 8.

In the context of pathogenic bacteria identification, immunosensors enable rapid screening of pathogens such as *Salmonella* and *Escherichia coli O157:H7* in dairy products. A comparison of the application of various immunosensors in pathogenic bacteria identification is presented as Table 9.

Immunosensors have formed a complete technological chain from laboratory validation to field application in dairy product testing. The main challenges currently faced include sensor stability in complex milk matrices, integration for simultaneous detection of multiple targets, and long-term preservation of sensor chips.

### 4.2. Microfluidics and Digital PCR Technologies

Microfluidics technology enables precise manipulation of small fluid volumes within micrometer-scale channels, achieving detection capabilities characterized by low sample consumption, fast analysis speed, and high integration [76]. For somatic cell counting, conventional methods (flow cytometry) require expensive equipment, while the California Mastitis Test, although simple, cannot provide quantitative results. The key performance indicators of various detection systems for somatic cell counting applications are presented as Table 10.

In the field of rapid microbial typing, representative technologies include: a triplex immunomagnetic separation coupled with microfluidic loop-mediated isothermal amplification (LAMP) technology, capable of simultaneous detection of *Escherichia coli O157:H7*, *Salmonella enteritidis*, and *Staphylococcus aureus* within 80 min with visual readout; a surface acoustic wave-driven microfluidic system that enables bacterial capture and detection from milk samples within 1 h, with a detection limit as low as 34 CFU/mL; and a paper-based microfluidic chip combined with aptamer-functionalized microspheres, completing detection within 15–20 min at a cost below $1.

Compared with traditional quantitative PCR (qPCR), microfluidic digital PCR (dPCR) technology offers several core advantages [80]. First, absolute quantification capability: dPCR partitions the reaction mixture into thousands to tens of thousands of independent micro-reactions, enabling direct calculation of target concentration via Poisson statistics without the need for a standard curve, thus eliminating quantification bias caused by standard curve variability. Second, high tolerance to PCR inhibitors: the partitioning strategy distributes inhibitors present in milk matrices (e.g., fat, proteins, calcium ions, and humic acids) unevenly across micro-reactions; most partition units remain inhibitor-free, and only those reactions containing both target DNA and inhibitors are affected, while the large number of unaffected positive reactions still provides reliable quantification. This makes dPCR 5–50 times more tolerant to common milk-derived inhibitors than qPCR. Third, low-abundance detection capability: digital signal output (positive/negative counting) significantly improves the signal-to-noise ratio, allowing reliable quantification of target copy numbers as low as 1–10 copies per reaction, which is particularly advantageous for detecting low-level contaminating pathogens and rare sequences [81]. Fourth, multiplexing potential: when combined with multi-color fluorescence channels and droplet generation technologies, dPCR enables simultaneous absolute quantification of 3–5 target pathogens in a single run, significantly reducing reagent consumption and assay time. Additionally, integrated microfluidic chips reduce sample and reagent consumption to nanoliter volumes, further decreasing costs and enabling high-throughput screening applications.

Since dPCR results depend on sample preparation and matrix, the limitations must be acknowledged. PMA-dPCR for quantifying viable L. monocytogenes, while effective under controlled conditions, is sensitive to milk fat and protein content, requiring matrix-specific optimization. Performance in naturally contaminated samples and detection limits are matrix-dependent, and PMA efficiency varies with milk type. The method relies on target-specific primers, limiting detection to selected pathogens, and requires specialized equipment unsuitable for field use. Thus, PMA-dPCR is best viewed as a research or high-level QC tool rather than a universal screening solution, with further validation on diverse dairy matrices needed before routine application.

## 5. Multi-Omics Approaches to Milk Composition

Multi-omics technologies enable panoramic profiling of milk components at the molecular level, overcoming the limitation of “single-parameter detection” inherent in traditional methods. They are applied to high-end applications such as quality traceability, functional discovery, health warnings, and process optimization, and mainly include metabolomics, proteomics, lipidomics, and microbiomics.

### 5.1. Metabolomics

Metabolomics aims to comprehensively analyze the complete set of small-molecule metabolites in biological samples [82]. Liquid chromatography-mass spectrometry (LC-MS), with its high separation capability, high sensitivity, and wide dynamic range, has become the core platform for constructing milk metabolic fingerprints. Based on different chromatographic separation mechanisms, A comparison of the two chromatographic separation approaches is presented as Table 11.

The introduction of ultra-high-performance liquid chromatography-ion mobility spectrometry-high-resolution mass spectrometry (UHPLC-IMS-HRMS) has brought new breakthroughs in the construction of milk metabolic fingerprints. Riboni et al. [84] applied UHPLC-IMS-HRMS for the first time to the traceability analysis of raw milk. The core advantage of this technology lies in the fact that ion mobility separation enables orthogonal separation based on the shape, charge, and mass of ions in the gas phase, significantly enhancing the discrimination capability for isomers and isobars.

LC-MS-based milk metabolic fingerprinting technology has achieved significant progress in the following directions [85]: geographical origin traceability and authenticity authentication (distinguishing samples from different sources based on metabolic profile differences); adulteration screening—Mung et al. successfully constructed metabolic fingerprints of human milk and five potential adulterants using a dansyl labeling LC-MS method, enabling the detection of as low as 5% exogenous milk adulteration [86]; physiological status monitoring (assessing energy metabolism and udder health); and fermentation process monitoring (tracking dynamic changes of flavor compounds).

The application of metabolic biomarkers in the early warning of dairy cow health status represents an important practical direction of metabolomics [87]. Ketosis is the most common metabolic disorder in periparturient dairy cows, characterized by the accumulation of ketone bodies (BHBA, acetoacetate, acetone) in blood and milk. Metabolomic studies have screened a series of novel biomarkers: Tumonoic acid H achieved 100% specificity for predicting ketosis 14 days prepartum, while LPE (18:0/0/0) and Ser-Leu-Lys-Ala-Ser reached 90% sensitivity 7 days prepartum [88]. Additionally, researchers have suggested that 2-piperidinecarboxylic acid may serve as an emerging indicator of subclinical ketosis [89]; seven metabolites, including decanoylcarnitine, histidine, and isoleucine, were found to be persistently elevated during ketosis.

The mechanistic basis for using metabolic biomarkers in early ketosis warning lies in negative energy balance (NEB) during the transition period (3 weeks pre– to postpartum). NEB triggers adipose lipolysis via hormone-sensitive lipase, releasing NEFA (>0.6 mmol/L), the earliest indicator detectable 1–2 weeks prepartum. Hepatic NEFA uptake exceeds complete β-oxidation due to insufficient oxaloacetate, leading to ketone body production (acetoacetate, BHBA, acetone). Ketogenesis outpaces peripheral utilization, causing accumulation: blood BHBA > 1.2 mmol/L indicates subclinical ketosis, >3.0 mmol/L clinical; milk BHBA > 0.15 mmol/L is the subclinical threshold [90]. Concurrently, insufficient propionate limits gluconeogenesis, causing hypoglycemia (<2.5 mmol/L) that further exacerbates lipolysis and ketogenesis via positive feedback [91].

Mastitis, another major dairy disease, imposes $35–50 billion annual global losses from direct (yield loss, discarded milk, treatment, culling) and indirect (quality penalties, processing impairment) costs. Subclinical mastitis (SCM), ~70% of cases, costs $200–300/cow/lactation. Metabolomics shows promise for early warning of both clinical and subclinical mastitis through non-invasive screening [92]. The mechanism involves intramammary infection triggering pro-inflammatory cytokines (TNF-α, IL-1β, IL-6), which increase vascular permeability, alter acute-phase protein synthesis, and perturb amino acid and lipid metabolism. These systemic changes are reflected in blood, milk, and urine metabolite profiles, enabling detection before clinical signs appear. Key biomarkers identified across different biofluids are summarized in Table 12.

Correlation studies have shown that BHBA levels in the blood of ketotic dairy cows are significantly positively correlated with non-esterified fatty acids (NEFA) and total bilirubin, and significantly negatively correlated with glucose. Combined detection of multiple metabolic biomarkers can improve the accuracy and timeliness of early warning.

Oligosaccharides and phospholipids are important functional components in milk, and are both characterized by complex structures, numerous isomers, and large variations in content. Targeted and untargeted analytical strategies each have their own focus and complement each other, depending on the need for known target discovery versus unknown exploration. The difficulty in oligosaccharide analysis lies in the coexistence of saccharides with different degrees of polymerization, the challenge of distinguishing isomers, and the lack of commercial standards for most of them [96]. Untargeted broad-spectrum screening using ultra-high-resolution mass spectrometry enables the identification of over 110 oligosaccharides in human milk and more than 50 in bovine milk in a single run [97]. Targeted isomer-resolved quantification based on UPLC-MS/MS-MRM technology achieves isomer-resolved quantification of 21 natural human milk oligosaccharides in just 12 min, with detection limits at the ng/L level. Pseudo-targeted analysis falls between these two approaches. A comparison of the three analytical modes is presented as Table 13.

### 5.2. Proteomics

Milk proteins mainly consist of casein and whey protein. These two protein classes exhibit significant differences in molecular structure, functional properties, thermal stability, and digestion kinetics [101]. The whey protein-to-casein ratio is a key quality control parameter in infant formula design. According to Codex standards, this ratio is typically required to be 60:40 for infant formula.

Proteomics based on high-resolution mass spectrometry provides a powerful tool for in-depth analysis of milk proteins. Through the identification of enzymatically digested peptides by LC-MS/MS, it enables the discrimination of casein and whey protein subtypes from different milk sources (bovine, caprine, camel, etc.). In adulteration identification, the screening of species-specific peptide markers can identify behaviors such as the adulteration of goat milk with cow milk or the adulteration of animal milk with plant proteins.

Post-translational modifications (PTMs) significantly influence the functional properties of milk proteins [102]. Phosphorylation primarily occurs on serine, threonine, and tyrosine residues. In human colostrum, 30 phosphorylation sites on 24 phosphoproteins were identified, whereas only nine phosphorylation sites on eight phosphoproteins were identified in mature milk. Phosphoproteins in colostrum are mainly involved in immune regulation and inflammatory responses. The degree of casein phosphorylation is a key factor affecting milk coagulation properties. Functional properties and effects of the Maillard reaction is presented as Table 14.

Pasteurization (72–85 °C for 15–30 s) and ultra-high temperature sterilization (UHT, 135–150 °C for 2–5 s) are the two most representative thermal processing techniques in the dairy industry. During heat treatment, denatured whey proteins form aggregates and subsequently cross-link with casein micelles. Methods for determining the whey protein denaturation rate can be classified into four major categories: immunological (ELISA), chromatographic (HPLC), spectroscopic (FTIR, CD), and fluorometric (FAST), as presented in the Table 15.

Pasteurization results in a whey protein denaturation rate of approximately 10–15%, with bioactive proteins still retaining partial activity. UHT treatment leads to a denaturation rate as high as 70–90%, accompanied by significant Maillard reactions. Advanced glycation end-products can be quantitatively assessed using the FAST index or furosine. A single marker is insufficient to cover the full range of heat treatment intensities; therefore, a combination of multiple heat markers (furosine, lactulose, native/denatured α-lactalbumin, FAST index, etc.) is required for comprehensive evaluation.

### 5.3. Lipidomics and Microbiomics

Triglycerides (TAG) account for 97–98% of total milk fat, and their fatty acid composition and positional distribution determine the nutritional functionality of milk fat. For detection, UPLC-Q-TOF-MS is currently the mainstream platform, enabling the separation, identification, and relative quantification of hundreds of TAGs in a single run.

Beyond routine TAG profiling, modern lipidomics based on high-resolution mass spectrometry has enabled the comprehensive characterization of milk lipid species at the molecular level [112]. Recent studies have identified over 400 distinct lipid species across multiple classes—including triglycerides, phospholipids (phosphatidylcholine, phosphatidylethanolamine, phosphatidylinositol), sphingolipids (sphingomyelin, ceramides), and gangliosides—in bovine milk using UHPLC-Q-TOF-MS and UHPLC-IMS-HRMS platforms [113]. This molecular-level resolution has revealed that the nutritional and functional properties of milk fat are determined not only by total fatty acid composition but also by the regioisomeric distribution of fatty acids on the glycerol backbone (sn-1, sn-2, sn-3 positions), which influences lipid digestion, absorption, and metabolic fate in humans [114].

Lipidomic profiling has also emerged as a powerful tool for authentication and quality control. Studies have demonstrated that lipid fingerprints can effectively distinguish milk from different species (bovine, caprine, ovine, buffalo), different production systems (organic vs. conventional, pasture-fed vs. grain-fed), and different geographical origins [115]. Furthermore, lipidomics has been applied to detect adulteration of high-value dairy products—such as goat milk, sheep milk, and buffalo milk mozzarella—with cheaper bovine milk or vegetable oils, achieving detection sensitivity as low as 1–5% adulteration through the identification of species-specific lipid markers.

In the context of dairy processing, lipidomic analysis has provided insights into the fate of milk lipids during thermal processing (pasteurization, UHT treatment), homogenization, and storage. For instance, oxidation of unsaturated fatty acids during storage generates oxidized triglyceride species and lipid oxidation products that correlate with off-flavor development and nutritional quality loss; these oxidative changes can be monitored through targeted lipidomic approaches to assess product shelf-life and quality stability [116]. Additionally, the milk fat globule membrane (MFGM)—a complex tri-layer structure rich in phospholipids, sphingolipids, and proteins—has received increasing attention for its bioactive properties and its role in stabilizing the fat emulsion; lipidomic characterization of MFGM components has enabled the development of MFGM-enriched ingredients for infant formula and functional foods.

In addition to lipid components, the microbial community present in milk is increasingly recognized as a critical factor influencing dairy quality, safety, and processing characteristics. While lipidomics focuses on the molecular profiling of fat components, microbiomics provides complementary information on the biological agents that contribute to spoilage, fermentation, and pathogenicity. The following section addresses the application of metagenomics and microbiomics in milk analysis [117].

Metagenomics, by directly extracting the genomic DNA of all microorganisms present in a sample and utilizing high-throughput sequencing technologies, enables comprehensive analysis of microbial community species composition, gene functions, and metabolic potential without the need for cultivation. The microbial community of raw milk from healthy cows is predominantly composed of lactic acid bacteria, *Streptococcus* spp., and *Propionibacterium* spp. Environmental exposure during milking introduces environment-associated microorganisms such as *Pseudomonas*, *Acinetobacter*, and *Escherichia coli.* Under mastitis conditions, the microbial diversity of raw milk significantly decreases, accompanied by overgrowth of pathogenic bacteria (e.g., *Staphylococcus aureus*, *Streptococcus agalactiae*). Dairy spoilage is mainly caused by psychrophilic bacteria, whose secreted heat-resistant proteases and lipases lead to age gelation and flavor deterioration in UHT milk during storage. Metagenomics enables the analysis of spoilage potential at the genetic level, identifying the types of degradation enzyme genes (aprX, serralysin, lipA/lipB, etc.) carried by spoilage bacteria.

Beyond species-level characterization, modern metagenomic approaches have evolved substantially in their application to dairy quality control. The adoption of shotgun metagenomics, as opposed to traditional 16S rRNA amplicon sequencing, provides direct insight into the functional genetic potential of microbial communities—including the presence of antibiotic resistance genes (the “resistome”), virulence factors, and metabolic pathways relevant to spoilage or fermentation [118]. This functional resolution is critical for distinguishing between harmless commensals and potentially pathogenic strains that share high sequence similarity at the 16S rRNA level.

In the context of dairy spoilage, metagenomic analysis has been applied to map the distribution of heat-stable enzyme genes among psychrophilic bacterial populations in raw milk. Studies have shown that the abundance of aprX (protease) and lipA/lipB (lipase) genes in raw milk metagenomes correlates with the subsequent shelf-life of UHT milk, enabling the prediction of age gelation and flavor deterioration risk at the point of raw milk reception [119]. This genotype-based risk assessment offers a significant advantage over traditional culture-based methods, which require extended incubation times and cannot capture the heat-stable enzyme production potential of psychrophilic populations.

Metagenomic-based source tracking has been employed to trace contamination routes in dairy processing environments. By comparing the microbial community composition of raw milk, processing line surfaces, and final products, researchers have distinguished resident processing plant microbiota from transient contaminants originating from farm environments or bulk transport. This approach has identified critical control points for hygiene management—such as milking equipment surfaces, bulk tank interiors, and heat exchanger plates—where targeted sanitation interventions can most effectively reduce contamination risks.

In the fermented dairy sector, metagenomics has revealed the complex microbial ecology underlying traditional products such as kefir, naturally fermented yogurts, and artisanal cheeses. Metagenomic sequencing has enabled the discovery of previously unculturable microbial species that contribute to flavor development, texture formation, and bioactive compound production in these products. These findings provide a scientific basis for starter culture optimization, quality standardization, and the preservation of traditional fermentation practices.

Furthermore, the application of metagenomics to mastitis diagnostics represents an emerging frontier. Conventional mastitis pathogen identification relies on culture-based methods that fail to detect approximately 10–30% of causative agents due to fastidious growth requirements or prior antibiotic treatment. Metagenomic sequencing of milk samples from mastitic quarters has enabled the detection of polymicrobial infections and the identification of emerging mastitis pathogens that are not captured by routine culture, offering the potential for more targeted and effective treatment strategies.

Metagenomics has evolved from species composition analysis to functional gene characterization and strain-level traceability. It not only enables comprehensive assessment of microbial diversity in raw milk but also identifies heat-stable spoilage enzyme genes and predicts the storage stability of UHT milk. Although the cost of sequencing and data analysis remains a barrier to widespread adoption, with decreasing sequencing costs and standardization of bioinformatics tools, metagenomics is expected to become a routine tool for microbiological quality control of dairy products.

## 6. Challenges and Key Scientific Issues

### 6.1. Complex Matrix Interference and Bottlenecks in Sample Pretreatment

Milk fat (3–5%) exerts a masking effect on trace contaminant detection far beyond its mass proportion, through three interference mechanisms:

(1) Physical entrapment—Fat globules form a hydrophobic core that partitions lipophilic contaminants (e.g., organochlorine pesticides, PCBs, dioxins), creating a “lipid trap.” Conventional liquid-liquid extraction fails to disrupt globule structures, markedly reducing analyte recovery.
(2) Co-extraction interference—Lipids are co-extracted with target analytes into the organic phase at concentrations orders of magnitude higher. This causes chromatographic column fouling (retention time drift, efficiency loss), ionization suppression in LC-MS/MS via charge competition, and detector contamination, increasing maintenance frequency.
(3) Detector response suppression—Thermal decomposition of lipids generates non-volatile residues that coat ion source surfaces, progressively reducing sensitivity.

The sample preparation bottleneck extends beyond fat interference to encompass the entire workflow from raw milk to instrument-ready extract. For oligosaccharide analysis, the enrichment and purification generally follow a progressive strategy of “defatting → deproteinization → lactose removal”, yet each step introduces potential losses. Defatting efficiency varies with temperature and centrifugation conditions; protein precipitation may co-precipitate target oligosaccharides through hydrophobic interactions; and enzymatic lactose removal is incomplete for highly polymerized oligosaccharides [120]. Similarly, for hormone analysis at ng/L levels, conventional solid-phase extraction cartridges offer insufficient enrichment factors, while novel sorbents (molecularly imprinted polymers, metal-organic frameworks) remain at the research stage with limited batch-to-batch reproducibility. The cumulative recovery loss across multi-step pretreatment—often 30–50% for trace components—represents a fundamental sensitivity constraint that cannot be fully compensated even by the most sensitive mass spectrometers.

Trace components in milk, such as oligosaccharides and hormones, typically account for less than 0.1% of the total mass and coexist with major matrix components, including milk fat, milk protein, and lactose. Direct analysis faces severe matrix interference and insufficient sensitivity [8]. The enrichment and purification of milk oligosaccharides generally follow a progressive strategy of “defatting → deproteinization → lactose removal.” Common methods include membrane separation, chromatographic separation, and enzymatic removal of lactose.

Hormones are present in milk at extremely low levels (ng/L to μg/L range), imposing high demands on the specificity and sensitivity of enrichment methods. Novel solid-phase adsorption materials, molecularly imprinted polymers, and magnetic dispersive solid-phase extraction are current research hotspots.

### 6.2. Lack of Reference Materials and Metrological Traceability

The prerequisite for accurate detection is the availability of traceable reference materials. However, in the dairy field, a large number of newly discovered functional components—particularly human milk oligosaccharides (HMOs), milk-derived bioactive peptides, and specific phospholipid subclasses—still lack commercial reference standards, or the available standards are extremely expensive and inconsistently supplied, severely restricting the development of quantitative detection methods and the comparability of results between laboratories.

The accessibility of reference standards for human milk oligosaccharides (HMOs) faces multiple obstacles: over 200 different oligosaccharide structures have been identified in human milk, but only a few (e.g., 2′-Fucosyllactose, 3-Fucosyllactosamine, Lacto-N-neotetraose, 3′-Sialyllactose,6′-Sialyllactose) are commercially available as reference standards produced via microbial fermentation or enzymatic synthesis; most oligosaccharides (especially those with high degrees of polymerization and complex branched structures) have no commercial sources; and numerous isomers exhibit highly similar physicochemical properties, requiring multi-step preparative chromatography purification to obtain high-purity single isomers [121].

The lack of reference standards for milk-derived bioactive peptides stems from several factors: the extremely high diversity of peptide sequences; bioactive peptides typically exist as mixtures rather than single compounds; the evaluation of “activity” relies on cell-based or animal experiments; and chemically synthesized peptides are costly and cannot fully replicate the true distribution of enzymatic hydrolysates [122].

The fatty acid composition of milk phospholipids is highly variable; a single phospholipid subclass can present dozens of molecular species depending on the esterified fatty acids. Quantification using a single molecular species standard cannot reflect the complete compositional profile of phospholipids in natural samples.

### 6.3. Metrological Validation and Standardization Challenges of Rapid Detection Methods

Rapid detection methods (immunochromatographic test strips, biosensors, portable spectrometers, etc.) are widely used in scenarios such as on-farm self-inspection and raw milk acceptance testing, but face numerous challenges in metrological validation and standardization.

In terms of metrological validation, the following dilemmas exist: most rapid detection methods provide only qualitative or semi-quantitative results, yet users often expect definitive judgments, leading to ambiguity in positioning; calibration relies on reference materials, but existing reference materials are mostly pure analyte solutions, which differ significantly from the complex composition of real milk matrices; the detection limit of visual readout methods depends on human visual acuity, introducing subjective variability.

The core challenges of standardization include: the diversity of methodological principles makes standardization difficult—immunochromatography, biosensors, portable spectroscopy, and microfluidic chips each have different key performance parameters and standardization hurdles; the lack of unified performance validation standards—the existing AOAC validation procedures do not adequately cover the unique attributes of rapid detection methods; and significant variation in regulatory acceptance of rapid detection methods across different countries and regions—the European Union classifies them as “screening methods”, the US FDA recognizes some methods for specific scenarios, and China is in the process of developing relevant technical specifications. The controversy over comparability between rapid methods and traditional methods also represents a major obstacle to standardization: how should discrepancies in detection results be adjudicated? How should acceptable error ranges be defined? The current consensus is that the performance of rapid methods should be no less than that of traditional methods, but this consensus is difficult to quantify at the implementation level.

Beyond technical hurdles, milk component analysis faces systemic challenges—economic, regulatory, and technical—that hinder translation of advanced methods into routine practice.

Economic viability. Multi-omics platforms (e.g., LC-HRMS) cost $200k–800k with 8–15% annual maintenance and $50–500/sample, versus $1–5 for FTIR or $10–20 for targeted LC-MS/MS. This creates a two-tier system: well-funded labs use omics, while most quality control labs rely on rapid screening. Even if omics could have detected the 2008 melamine crisis earlier, such instruments were absent from production-line settings, highlighting the need for affordable, fit-for-purpose solutions.

Data interpretation gap: A single LC-HRMS run yields 5000–10,000 features, but few are confidently identified. Statistical significance often lacks practical meaning; many discriminatory features lack chemical standards for confirmation. Machine learning offers promise but suffers from scarce annotated datasets, poor interpretability, and “black-box” concerns for regulatory validation.

Supply chain harmonization: Dairy supply chains involve diverse stakeholders using different methods, instruments, and data formats. Inconsistent FTIR calibration models, lack of conversion between qualitative screening and quantitative confirmatory results, and variable reporting standards complicate cross-laboratory comparison and international trade.

Workforce skills gap: The shift from wet chemistry to omics demands expertise in MS, bioinformatics, and chemometrics, but university curricula lag. This leads to instrument downtime, inconsistent data quality, underutilized advanced instruments, and staff retention challenges.

Regulatory adaptation lag: Validation protocols were designed for slow-evolving methods, but today’s rapid innovation outpaces regulatory recognition—often by years. Priorities include performance-based criteria, expedited approval pathways for equivalent new technologies, international mutual recognition agreements, and guidelines for multi-analyte and untargeted omics methods.

## 7. Summary and Discussion

This paper reviews the evolution of milk component detection technologies, from traditional classical methods to cutting-edge multi-omics approaches, covering basic physicochemical detection methods, chromatographic techniques, infrared spectroscopy, biosensing and microfluidic technologies, as well as modern analytical platforms such as metabolomics, proteomics, lipidomics, and microbiomics. Through a systematic comparison of the principles, advantages, limitations, and applicable scenarios of each technological platform, the following core findings and development trends can be summarized.

The development of milk component detection technologies exhibits a clear trajectory: from “single-parameter” to “multi-parameter”, from “off-line” to “on-line”, and from “laboratory” to “on-site” [123]. First, detection throughput and information dimensions continue to improve. Traditional methods such as the Kjeldahl method and Soxhlet extraction yield only a single parameter per experiment and require hours to days. Modern UHPLC-MS/MS platforms can simultaneously determine dozens or even hundreds of compounds in a single run, while metabolomics and proteomics further extend the detection dimension from “quantification” to “panoramic profiling” [124]. Second, detection speed and convenience have significantly increased. Traditional methods involve cumbersome procedures and rely on complex sample pretreatment, whereas infrared spectrometers can complete simultaneous multi-component quantification within seconds, and immunochromatographic test strips and handheld biosensors have made on-site screening possible. Third, analytical depth has extended from component quantification to molecular structure elucidation. Traditional methods can only determine total protein or total fat content, whereas modern mass spectrometry techniques can distinguish β-casein A1/A2 variants, identify glycosidic linkage patterns of oligosaccharides, and locate phosphorylation/glycosylation modification sites on proteins.

By application scenario: (1) Laboratory-based reference methods (e.g., Kjeldahl, Dumas, Röse–Gottlieb, HPLC-UV, ICP-MS) serve as gold standards for regulatory compliance and arbitration, offering high accuracy but are time-consuming and labor-intensive. (2) High-throughput routine screening platforms (e.g., FTIR/MIR in DHI, UHPLC-MS/MS) balance speed and reliability for batch processing in centralized labs, widely used for payment systems and herd health monitoring. (3) On-site rapid detection tools (e.g., immunochromatographic strips, portable biosensors, microfluidic chips) prioritize portability and speed over absolute accuracy, enabling real-time decision-making at farms, plants, and market checkpoints.

By technology type: (1) Physicochemical methods (total nitrogen, fat extraction, basic physicochemical tests) are robust and standardized but provide limited molecular details. (2) Separation-based instrumental methods (chromatography, electrophoresis hyphenated to MS) offer high separation power and sensitivity for targeted components (vitamins, oligosaccharides, fatty acids) but require extensive sample preparation. (3) Spectroscopic methods (MIR, NIR, Raman, fluorescence) are rapid and non-destructive, ideal for high-throughput screening, yet depend on continuously validated chemometric models. (4) Biosensing and molecular diagnostic methods (enzyme-based biosensors, immunosensors, digital PCR) offer specificity and multiplexing potential, but face challenges in sensor stability, matrix interference, and batch-to-batch reproducibility.

By detection target: (1) Macronutrients (fat, protein, lactose, solids) are routine parameters with well-established methods. (2) Micronutrients and functional components (vitamins, minerals, oligosaccharides, bioactive peptides) require sophisticated techniques (LC-MS, ICP-MS) due to low concentrations and structural diversity, driven by nutritional labeling and quality differentiation. (3) Hazardous risk factors (antibiotic residues, mycotoxins, heavy metals, pathogens, adulterants, SCC, BHBA) are safety-critical with stringent limits, demanding multi-analyte screening and early-warning capabilities.

These three dimensions provide a decision-making framework for method selection and highlight gaps—especially the need for field-deployable multi-analyte platforms and standardized protocols enabling cross-method comparability.

Key scientific issues: (1) Matrix interference and pretreatment bottlenecks—fat co-extraction remains a challenge; future work should develop novel adsorbents and on-line pretreatment-detection systems. (2) Lack of reference materials and metrological traceability—promote the industrialization of standards, develop standard-free quantification, and build shared spectral libraries. (3) Standardization challenges for rapid methods—there is an urgent need for performance validation guidelines clarifying detection limits and acceptable false-positive rates. (4) Biological interpretation of multi-omics data—many signals lack structural assignment; causal relationships with milk quality need validation. Efforts should integrate machine learning to improve biomarker screening efficiency and link omics data to physiological indicators.

The landscape of milk component analysis has evolved from labor-intensive classical methods to high-throughput instrumental platforms and, more recently, to multi-omics approaches. However, the translation of these advances into routine dairy quality control remains uneven, with a clear divide between well-funded reference laboratories and production-line testing facilities.

Priority research directions for the coming 5–10 years:

(1) Field-deployable multi-analyte platforms. Portable, low-cost devices for the simultaneous detection of multiple components and contaminants remain a critical unmet need, particularly for decentralized dairy supply chains in developing countries. Microfluidic chips, paper-based sensors, and smartphone-integrated systems require further validation under real-world field conditions.
(2) AI and machine learning integration. The vast data generated by FTIR and multi-omics platforms remains underutilized. AI-enhanced spectral interpretation, automated feature selection, and predictive modeling offer significant opportunities for improving the speed and accuracy of milk analysis.
(3) Reference material development. The persistent lack of commercial standards for HMOs, bioactive peptides, and specific lipids requires coordinated efforts between academia, industry, and regulators. Standard-free quantification strategies and shared spectral libraries may provide interim solutions.
(4) Standardization of rapid methods. The absence of unified validation standards for rapid detection methods (biosensors, immunochromatographic strips, portable NIR) creates uncertainty in regulatory acceptance. International consensus on validation protocols and equivalence criteria is urgently needed.
(5) Integration of omics with traditional quality parameters. Multi-omics approaches generate vast data with the potential to transform milk quality assessment, yet their integration with established parameters (fat, protein, SCC) remains limited. Validated correlations between omics signatures and practical quality outcomes are needed to enable biomarker-based prediction models.

Concluding remarks. Milk component analysis is at an inflection point where classical methods, instrumental platforms, and multi-omics technologies coexist and complement each other. No single technology is universally optimal; the choice depends on the specific target, scenario, resources, and throughput requirements. The future lies not in replacing established methods with novel technologies, but in developing integrated, context-appropriate strategies that leverage multiple platforms. Bridging the gaps between innovation and implementation—addressing technical performance, economic viability, workforce capacity, and regulatory adaptation—will be critical to achieving the dual goals of product safety and quality while meeting consumer demand for transparency and nutritional excellence.

## Figures and Tables

**Table 1:** Detection strategies for micronutrients and functional components.

Component Category	Detection Methods	Key Technical Characteristics	LOD/LOQ
Vitamins (Water-soluble) [23]	UHPLC-MS/MS, Microbiological method	Ion-pair chromatography, Derivatization treatment	0.1–5 μg/L (LC-MS); 0.1–1 μg/L (microbiological)
Vitamins (Fat-soluble) [24]	HPLC-UV/DAD, UHPLC-MS/MS	Saponification extraction, Normal-phase/reversed-phase separation	1–50 μg/L (HPLC-UV); 0.1–5 μg/L (UHPLC-MS/MS)
Minerals and Trace Elements [25]	ICP-OES, ICP-MS	Microwave digestion pretreatment, Simultaneous determination of multiple elements	0.1–10 μg/L (ICP-MS); 1–100 μg/L (ICP-OES)
Oligosaccharides [26]	HPAEC-PAD, HILIC-MS, CE-LIF	Requires removal of protein/lactose interference and enrichment prior to detection	0.1–5 mg/L (HPAEC-PAD); ng/L level (UHPLC-MS/MS)
Enzyme Activity [27]	Fluorogenic substrate method, UV-Vis	Uses activity rather than content as the detection endpoint	Activity-dependent (μmol/min/mL)

**Table 2:** Kjeldahl vs. Dumas method vs. Gerber method: A comparison.

Method	Advantages	Limitations
Kjeldahl method [31]	Good accuracy and reproducibility; wide adaptability to samples with different matrices	Cumbersome operation and time-consuming; use of hazardous chemicals such as concentrated sulfuric acid; unable to distinguish between protein nitrogen and non-protein nitrogen
Dumas method [32]	Fast analysis speed; high degree of automation; no use of toxic chemical reagents	Likewise unable to distinguish between protein nitrogen and non-protein nitrogen; determination results tend to be overestimated in samples with low protein content

**Table 3:** Soxhlet vs. Röse–Gottlieb: Limitations and improvements.

Method	Limitations	Improvement Strategies
Soxhlet extraction method [34]	Lengthy operation cycle; high solvent consumption; not suitable for high-moisture samples	Automated acid hydrolysis-Soxhlet extraction coupled system: reduces manual operation time to approximately 2 h
Röse–Gottlieb method [35]	Low extraction efficiency for low-fat matrices (e.g., human milk)	Solid-phase extraction-based improvement strategy to enhance fat extraction efficiency from complex matrices
Gerber method [35]	Lower accuracy than gravimetric methods; sulfuric acid safety hazard; not suitable for low-fat/fermented products; operator-dependent; requires sample-specific correction factors	Rapid (5–10 min); no drying/pre-extraction; simple equipment; low cost; suitable for routine quality control in processing plants

**Table 4:** HPAEC-PAD vs. UHPLC-MS: Advantages for lactose and oligosaccharides.

Parameter	HPAEC-PAD [37]	UHPLC-MS [36]
No derivatization required	Yes	Yes
Ease of operation	High	Relatively high; mass spectrometry requires maintenance
Isomer discrimination capability	Moderate	Strong
Structural information acquisition	None	Rich (molecular mass, fragmentation patterns, etc.)
Run time per sample	20–40 min	8–15 min (UHPLC); 20–45 min (conventional HPLC-MS)
LOD	0.1–10 μg/L	0.01–1 ng/L for HMOs
LOQ	1–50 μg/L	0.05–5 ng/L

**Table 5:** Technical comparison and application division between MIR and NIR spectroscopy.

Dimension	MIR [41,42]	NIR [43,44]
Spectral Origin	Fundamental vibrations	Overtones and combination vibrations
Absorption Intensity	Strong	Weak
Spectral Characteristics	Sharp peaks, strong characteristic features	Broad, overlapping peaks, difficult to resolve
Sample Penetration Depth	Micrometer level	Millimeter to centimeter level
Pretreatment Requirements	Requires homogenization	Relatively relaxed
Quantification Approach	Direct application of the Lambert–Beer law	Relies on chemometric models
Primary Applications	Precise quantification of routine components	Adulteration screening, solid sample analysis

**Table 6:** Impact of milk components determined by FTIR spectral data on health monitoring.

Component	Detected Concentration Range	Health Status
β-Hydroxybutyric acid [49]	0.05–1.0 mmol/L	Clinical/subclinical ketosis
Acetone [50]	0.02–0.5 mmol/L	Ketosis (confirmatory marker)
Lactoferrin [51]	0.1–10 mg/L	Udder inflammation (mastitis)
Progesterone [52]	0.5–20 ng/mL	Pregnancy status
Fatty acids [53]	0.1–30 g/100 g fat	Rumen function; energy metabolism
Lactose [54]	4.2–5.0%	Udder health; nutritional status
Total protein [55]	3.0–4.5%	Nutritional status; metabolic health
SCC [56]	50,000–2,000,000 cells/mL	Udder health; mastitis

**Table 7:** Application of various enzyme-based biosensors in lactose detection.

Sensor Type	Enzyme System	Principle	Key Performance	Limitations
Cascade enzyme sensor [59]	β-Gal + GOx	Lactose → Glucose + H_2_O_2_	Detection limit: 0.036 mM	Complex system, relatively high cost
Single-enzyme sensor [60]	LOx	Lactose → Lactobionic acid + H_2_O_2_	Detection limit: 1.4 μM	Maturity needs to be improved
Dehydrogenase-based sensor [61]	β-Gal + GDH	Lactose → Glucose → NADH	Zero-potential operation, strong anti-interference capability	Requires dual-sensor calibration

**Table 8:** Application of various immunosensors in the detection of milk source protein adulteration.

Sensor Type	Detection Target	Assay Time	Characteristics
Prussian blue nanoparticle colorimetric immunosensor [65]	Bovine IgG	120 min	Enzyme-free, low cost, maintains >94% activity over 4 weeks
Label-free electrochemical immunosensor [66]	Bovine IgG	15 min (one-step)	Portable, wireless detection, coefficient of variation <6%
Mach–Zehnder interferometer photonic sensor [67]	Bovine κ-casein	12 min	Label-free, no pretreatment required, regenerable

**Table 9:** Application of various immunosensors in pathogenic bacteria identification.

Sensor Type	Detection Target(s)	LOD (CFU/mL)	Assay Time	Main Characteristics
Portable photonic immunosensor [68]	*Salmonella/* *Escherichia coli*	45/125	15 min	Simultaneous detection of dual pathogens, reusable for 10 cycles
NMR immunosensor [69]	*Salmonella*	740	90 min	RSD = 2.35%; high sensitivity
Electrochemical impedance immunosensor [70]	*L. monocytogenes*	16	60 min	Label-free; portable; suitable for on-site testing
Colorimetric lateral flow immunosensor [71]	*E. coli O157:H7*	1.0 × 10^3^	15 min	Cost-effective; visual readout; no equipment required
Fluorescence immunosensor [72]	*Salmonella/* *S. aureus*	50	45 min	Simultaneous detection; magnetic enrichment improves sensitivity
Surface-enhanced Raman scattering (SERS) immunosensor [73]	*L. monocytogenes*	10	30 min	Ultra-high sensitivity; multiplexing potential
Quartz crystal microbalance (QCM) immunosensor [74]	*E. coli O157:H7*	200	20 min	Real-time monitoring; label-free; regenerable
Microfluidic chip-integrated immunosensor [75]	Multiple pathogens (3-plex)	10–100	35 min	High integration; low sample volume

**Table 10:** Key performance indicators of various detection systems for somatic cell counting applications.

Detection System	Technical Principle	Assay Time	Key Performance
Handheld counter [77]	Microfluidic chip + fluorescence staining + image recognition	2 min	Error < 10%, accuracy 93%
mCytoCounter system [78]	Membrane filtration enrichment + fluorescence reading	15 min	R^2^ = 0.97 (vs. microscopy)
Smartphone platform [79]	Disposable chip + mobile phone optics	2 min	Cost < $1 per test

**Table 11:** Technical routes of the two chromatographic separation approaches.

Platform	Separation Mechanism	Applicable Metabolite Classes	Characteristics
RPLC-MS	Hydrophobic interaction	Non-polar to medium-polarity metabolites	Most widely applied, high peak capacity
HILIC-MS [83]	Polar interaction	Highly polar metabolites	Orthogonally complementary to RPLC

**Table 12:** Early-warning biomarkers for mastitis.

Biomarker	Performance
Blood biomarkers [93]	Significantly elevated levels of valine, serine, tyrosine, and phenylalanine in the serum of diseased cows
Milk biomarkers [94]	Decreased levels of glucose, D-glycerol-1-phosphate, etc., and increased levels of arginine and leucyl-leucine dipeptide in the milk of cows with CM
Urine biomarkers [95]	Abnormalities in symmetric dimethylarginine (SDMA), methylmalonic acid, etc., appearing 8 weeks before the onset of SCM

**Table 13:** Comparison and application selection of untargeted, pseudo-targeted, and targeted strategies.

Dimension	Untargeted [98]	Pseudo-Targeted [99]	Targeted [100]
Applicable Scenario	Unknown screening, panoramic profiling of the spectrum	Efficient screening of specific functional lipids	Accurate quantification of known components
Coverage	Hundreds to thousands of metabolites	Hundreds of specific lipid classes	Dozens of target compounds
Quantification Capability	Semi-quantitative/relative quantification	Relative quantification	Absolute quantification
Typical Technique	UHPLC-HRMS + FBMN	IDA + feature matching algorithm	UPLC-MS
Advantage	Panoramic information, discovery of novel structures	Balances coverage and specificity	High sensitivity, high accuracy
Limitation	Variable identification confidence	Relies on prior knowledge base	Unable to discover unknowns

**Table 14:** Functional properties and effects of the Maillard reaction.

Functional Property	Positive Impact	Potential Negative Impact
Thermal stability [103]	Improves the thermal stability of whey protein	Leads to loss of protein functionality
Emulsifying property [104]	Enhances the emulsifying activity and emulsion stability of whey protein	None reported
Solubility [103]	Significantly improves the solubility of whey protein at pH 5	None reported
Antioxidant activity [105]	Antioxidant activity increases with the degree of reaction	None reported
Allergenicity [106]	Reduces the allergenicity of dairy products	Associated with chronic diseases
Micellar structure [107]	The higher the degree of glycosylation, the smaller the casein micelle size	Affects milk stability and processing characteristics

**Table 15:** Parameters of different methods for determining whey protein denaturation rate.

Method	Principle	Discrimination Capability	Time	Application
ELISA [108]	Conformation-specific antibody recognition of native protein	High	2–4 h	Quantification of low-abundance proteins
HPLC [109]	Chromatographic quantification of soluble protein	High	1–2 h	Simultaneous quantification of multiple components
FTIR [110]	Spectral analysis of amide band secondary structure	Moderate	Real-time	Real-time monitoring of thermal denaturation process
CD [111]	Quantification of secondary structure in the far-UV region	Moderate	30 min	Secondary structure analysis in dilute solutions
FAST [109]	Dual fluorescence of tryptophan + Maillard reaction products	High	5 min	Rapid process differentiation and nutritional evaluation

## Data Availability

No new data were created or analyzed in this study. Data sharing is not applicable to this article.

## Supplementary Materials

The following supporting information can be downloaded at: https://www.mdpi.com/article/10.3390/foods15152752/s1, Table S1: Summary of key characteristics of the cited literature included in this review, including detection target, analytical technique, matrix, LOD/LOQ, and key performance parameters. References are numbered as in the main text.
