# Supplementary material for: Analytical Technologies for Milk Component Analysis: A Review of Progress and Challenges
Source: Foods. 2026 Aug 5;15(15):2752. doi: 10.3390/foods15152752 (PMC13465885; doi:10.3390/foods15152752)
Supplement: Supplementary file 1 [file foods-15-02752-s001.zip › foods-4432644-supplementary.pdf]

Supplementary Table S1. Summary of key characteristics of cited literature

No.	Reference	Detection Target	Analytical Technique	Matrix	LOD/LOQ	Key Performance	Year
**Chromatographic Methods**							
1	Acquavia et al. [8]	Milk components (review)	HPLC, UHPLC-MS/MS	Various dairy matrices	—	Comprehensive review of extraction and analytical methods	2025
2	Liu et al. [36]	Milk oligosaccharides	UPLC-MS/MS	Infant formula	ng/L level	Isomer-resolved quantification of 21 HMOs within 12 min	2023
3	van Scheppingen et al. [37]	Lactose	HPAEC-PAD	Low-lactose dairy products	—	Selective and sensitive determination	2017
4	Guo et al. [38]	Polar metabolites	HILIC	Various	—	Survey of polar stationary phases for HILIC	2022
5	Ji et al. [39]	Mare milk adulteration	LC-HRMS (proteomics + metabolomics)	Mare milk, cow milk	—	Species-specific peptide and metabolite markers	2023

No.	Reference	Detection Target	Analytical Technique	Matrix	LOD/LOQ	Key Performance	Year
6	Wang et al. [40]	Nucleosides and nucleotides	HPLC, LC-MS	Infant formula, milk	—	Analytical strategies for nucleosides/nucleotides	2025
7	Blake et al. [23]	Water-soluble vitamins	UHPLC-MS/MS, Microbiological	Foods, dairy	0.1–5 µg/L (LC-MS); 0.1–1 µg/L (microbiological)	Derivatization required; assays >24 h	2007
8	Plozza et al. [24]	Fat-soluble vitamins	HPLC-UV/DAD, UHPLC-MS/MS	Cow milk	1–50 µg/L (HPLC-UV); 0.1–5 µg/L (UHPLC-MS/MS)	Saponification extraction; normal/reversed-phase separation	2017
9	Lee et al. [25]	Minerals and trace elements	ICP-OES, ICP-MS	Milk	0.1–10 µg/L (ICP-MS); 1–100 µg/L (ICP-OES)	Microwave digestion; simultaneous multi-element determination	2022
10	Khan et al. [27]	Enzyme activity (protease, lipase)	Fluorogenic substrate, UV-Vis	Milk	Activity-dependent (µmol/min/mL)	Activity-based detection endpoint	2025
**Spectroscopic Methods**							

No.	Reference	Detection Target	Analytical Technique	Matrix	LOD/LOQ	Key Performance	Year
11	Loudiyi et al. [9]	Functional properties of milk	MIR, NIR, Raman, Fluorescence	Raw bovine milk	—	Spectroscopic methods with chemometric modeling	2022
12	Grelet et al. [41]	Milk composition	MIR spectroscopy	Bovine milk	—	Large-scale phenotyping using MIR spectra	2021
13	Morelli et al. [42]	Milk composition	MIR spectroscopy	Milk	—	Chemometric techniques for MIR spectral prediction	2025
14	Pu et al. [43]	Milk, cheese, dairy powders	NIR spectroscopy	Dairy products	—	Portable and handheld NIR spectrometers	2021
15	Uusitalo et al. [44]	Raw milk composition	MEMS NIR spectroscopy	Raw milk	—	On-farm analysis evaluation	2021
16	De Marchi et al. [45]	Milk traits	Mid-infrared spectroscopy	Milk	—	Phenotyping tool for milk traits (review)	2014
17	Tiplady et al. [46]	Milk traits	FTIR spectroscopy	Bovine milk	—	Genetic improvement of dairy cattle	2020
18	Yakubu et al. [47]	Dairy production	NIR spectroscopy	Dairy products	—	Review of NIR spectroscopy in dairy	2022

No.	Reference	Detection Target	Analytical Technique	Matrix	LOD/LOQ	Key Performance	Year
						production	
19	Saji et al. [48]	Dairy products	FTIR spectroscopy	Dairy products	—	Systematic review of FTIR applications	2024
20	Grelet et al. [49]	Acetone, BHBA, citrate	FTIR spectroscopy	Bovine milk	0.05–1.0 mmol/L (BHBA); 0.02–0.5 mmol/L (acetone)	European dairy network calibration	2016
21	Van Knegsel et al. [50]	Ketone bodies	FTIR spectroscopy	Milk	0.05–1.0 mmol/L (BHBA)	Detection of hyperketonemia in dairy cows	2010
22	Soyeurt et al. [51]	Lactoferrin	FTIR spectroscopy	Bovine milk	0.1–10 mg/L	Potential indicator of mastitis	2012
23	Tiplady et al. [52]	Progesterone	FTIR spectroscopy	Milk	0.5–20 ng/mL	Pregnancy status prediction	2022
24	Franceschini et al. [53]	Fatty acids	FTIR spectroscopy	Milk	0.1–30 g/100g fat	Detection of herd management issues	2025
25	Pyörälä et al. [54]	Lactose (inflammation indicator)	FTIR spectroscopy	Milk	4.2%–5.0%	Indicator of inflammation in mastitis diagnosis	2003

No.	Reference	Detection Target	Analytical Technique	Matrix	LOD/LOQ	Key Performance	Year
26	Giannuzzi et al. [55]	Milk phenomics	FTIR spectroscopy	Milk	3.0%–4.5% (protein)	Nutritional and health status evaluation	2024
27	Rienesl et al. [56]	Somatic Cell Count (SCC)	FTIR spectroscopy	Milk	50,000–2,000,000 cells/mL	Prediction of acute and chronic mastitis	2022
**Biosensing Methods**							
28	Wijayanti et al. [11]	Lactose	Enzyme-based biosensors	Milk	—	Review of electrochemical enzyme-based biosensors	2023
29	Fernández González et al. [16]	Dairy safety	Immunosensors	Dairy products	—	Progress and challenge of sensors for dairy safety	2024
30	Zhang et al. [57]	Foodborne pathogenic bacteria	Microfluidic biosensors	Milk	—	Review of microfluidic biosensors for pathogen detection	2025
31	Farag et al. [58]	Milk contaminants	Biosensors	Milk	—	Comprehensive review of biosensing approaches	2021

No.	Reference	Detection Target	Analytical Technique	Matrix	LOD/LOQ	Key Performance	Year
32	Han et al. [59]	Lactose	Cascade enzyme sensor (β-Gal+GOx)	Milk	0.036 mM	Complex system, relatively high cost	2013
33	Halpin et al. [60]	Lactose	Single-enzyme sensor (LOx)	Dairy samples	1.4 μM	Maturity needs to be improved	2019
34	Glithero et al. [61]	Lactose	Dehydrogenase-based sensor (β-Gal+GDH)	Dairy processing	—	Zero-potential operation, dual-sensor calibration	2013
35	Yu et al. [19]	Species identification	Immunosensors	Milk	—	Advancements in species identification	2024
36	Caroli et al. [62]	Milk protein polymorphisms	Immunosensors	Bovine milk	—	Effect on breeding and human nutrition	2009
37	Krishna et al. [63]	Milk proteins	Immunosensors	Milk	—	Influence of ultra-heat treatment on proteins	2021
38	Ionescu et al. [64]	Milk frauds	Immunosensors	Milk	—	Review of milk frauds and adulterations	2023
39	Farka et al. [65]	Food contaminants	Prussian blue nanoparticle immunosensor	Various	—	Bovine IgG detection, enzyme-free, >94% activity over 4 weeks	2017

No.	Reference	Detection Target	Analytical Technique	Matrix	LOD/LOQ	Key Performance	Year
40	Han et al. [66]	Food adulteration	Label-free electrochemical immunosensor	Milk	—	Bovine IgG, portable, wireless, CV <6%	2026
41	Kourti et al. [67]	Milk adulteration	Mach-Zehnder interferometer photonic sensor	Ewe, goat, donkey milk	—	Bovine κ-casein, label-free, regenerable, 12 min	2024
42	Kourti et al. [68]	Salmonella/E. coli	Portable photonic immunosensor	Milk	45/125 CFU/mL	Simultaneous detection of dual pathogens, 15 min	2023
43	Guo et al. [69]	Salmonella	NMR immunosensor	Milk	740 CFU/mL	Gd chelation signal amplification, RSD=2.35%, 90 min	2024
44	Lee et al. [70]	L. monocytogenes	Electrochemical impedance immunosensor	Milk	16 CFU/mL	Label-free, portable, 60 min	2022
45	Zhang et al. [71]	E. coli O157:H7	Colorimetric lateral flow immunosensor	Milk	1.0×10^3 CFU/mL	Cost-effective, visual readout, 15 min	2023
46	Cheng et al. [72]	Salmonella/S. aureus	Fluorescence immunosensor	Milk	50 CFU/mL	Simultaneous detection, magnetic enrichment, 45	2023

No.	Reference	Detection Target	Analytical Technique	Matrix	LOD/LOQ	Key Performance	Year
						min	
47	Liu et al. [73]	L. monocytogenes	SERS immunosensor	Milk	10 CFU/mL	Ultra-high sensitivity, 30 min	2025
48	Shen et al. [74]	E. coli O157:H7	QCM immunosensor	Milk	200 CFU/mL	Real-time monitoring, label-free, 20 min	2011
49	Xiang et al. [75]	Multiple pathogens	Microfluidic chip-integrated immunosensor	Milk	10-100 CFU/mL	High integration, low sample volume, 35 min	2023
**Microfluidic and Molecular Methods**							
50	Ng et al. [13]	Contamination detection	Microfluidic devices	Milk	—	Review of microfluidic devices for milk quality	2021
51	Lonchamps et al. [76]	Pathogens	Microfluidic lab-on-chip	Foods	—	Mini-review of pathogen detection	2022
52	Düven et al. [77]	Somatic cell count	Handheld microfluidic counter	Milk	—	Error <10%, accuracy 93%, 2 min	2019
53	Khaware et al.	Somatic cell	mCytoCounter system	Milk	—	R^2=0.97 (vs.	2025

No.	Reference	Detection Target	Analytical Technique	Matrix	LOD/LOQ	Key Performance	Year
	[78]	count				microscopy), 15 min	
54	Sun et al. [79]	Somatic cell count	Smartphone platform	Milk	—	Disposable chip + phone optics, <$1/test, 2 min	2024
55	Li et al. [80]	Adulteration identification	Digital PCR	Dairy products	—	Comparison of PCR techniques	2023
56	Hindson et al. [81]	DNA copy number	Droplet digital PCR	Various	—	High-throughput absolute quantification	2011
**Multi-Omics Methods**							
57	Rocchetti et al. [82]	Milk quality and traceability	Metabolomics (LC-MS)	Milk	—	Review of metabolomics applications	2021
58	Tang et al. [83]	Metabolomics	HILIC-MS	Various	—	HILIC-MS for metabolomics review	2016
59	Riboni et al. [84]	Raw milk traceability	UHPLC-IMS-HRMS	Raw milk	—	First application to raw milk traceability	2025
60	Becchi et al.	Milk quality	Multi-omics +	Milk	—	Integrated analytical	2025

No.	Reference	Detection Target	Analytical Technique	Matrix	LOD/LOQ	Key Performance	Year
	[85]		machine learning			approaches	
61	Mung et al. [86]	Milk adulterant detection	Dansyl labeling LC-MS	Human milk	5% exogenous milk adulteration	Chemical isotope labeling metabolomics	2018
62	Heirbaut et al. [87]	Metabolic health	Metabolomics	Milk	—	Diagnostic milk biomarkers for health status	2023
63	Ping et al. [88]	Ketosis	Multiomics	Dairy cow blood	—	Blood differential metabolites and genes	2024
64	Zhao et al. [89]	Cow health	Metabolomics	Various	—	Review of metabolomics in cow health	2025
65	Mondal et al. [90]	Subclinical ketosis	Metabolomics	Milk, blood	BHBA >0.15 mmol/L (milk)	Endocrine-metabolic regulation review	2026
66	Qiao et al. [91]	Insulin resistance	Metabolomics	Transition cows	—	Insulin resistance and metabolic inflammation	2024
67	Hu et al. [92]	Mastitis	Metabolomics	Milk	—	Review of metabolomics in mastitis diagnosis	2021

No.	Reference	Detection Target	Analytical Technique	Matrix	LOD/LOQ	Key Performance	Year
68	Zhang et al. [93]	Subclinical mastitis	Metabolomics	Serum	—	Serum-predictive biomarkers for SCM	2022
69	Du et al. [94]	Mastitis	Metabolomics	Milk	—	Milk metabolite profiling in mastitis	2024
70	Zwierzchowski et al. [95]	Subclinical mastitis	Metabolomics	Urine	—	Urinary metabotyping for SCM screening	2020
71	Zhu et al. [96]	Human milk oligosaccharides	LC-MS	Human milk	—	Critical review on HMO structure and analysis	2023
72	Perez de Souza et al. [97]	Metabolomics	UHPLC-HRMS	Various	—	Ultra-high-performance LC-HRMS variants	2021
73	Xi et al. [98]	Mastitis	Untargeted metabolomics	Milk	—	UPLC-Q-TOF-MS based untargeted milk metabolomics	2017
74	Li et al. [99]	Lipidomics	Pseudo-targeted lipidomics	Yak milk	—	UHPLC-Qtrap-MS based pseudo-targeted analysis	2024
75	Zhang et al. [100]	Milk diversity	Widely targeted metabolomics	Goat, sheep, cow, buffalo	—	Metabolomic analysis of milk from different	2024

No.	Reference	Detection Target	Analytical Technique	Matrix	LOD/LOQ	Key Performance	Year
				milk		species	
76	Nayik et al. [101]	Milk proteins	Proteomics	Milk	—	Milk proteins chemistry and functionality	2024
77	Scheuerer et al. [102]	Post-translational modifications	Proteomics	Milk proteins	—	PTMs and techno-functional properties	2026
78	Wang et al. [103]	Whey protein	Maillard reaction analysis	Whey protein	—	Glycosylation effects on solubility and stability	2012
79	Hou et al. [104]	Whey protein-pectin conjugates	Glycosylation analysis	Whey protein	—	Emulsifying ability evaluation	2024
80	Stobiecka et al. [105]	Antioxidant activity	Antioxidant activity assay	Milk and dairy products	—	Antioxidant activity of milk and dairy products	2022
81	Toda et al. [106]	Allergenicity	Glycosylation analysis	Milk proteins	—	Maillard reaction and allergenicity	2019
82	Runthala et al. [107]	Casein micelle structure	Proteomics	Milk	—	Casein micellar organization and	2023

No.	Reference	Detection Target	Analytical Technique	Matrix	LOD/LOQ	Key Performance	Year
						functionality	
83	Lu et al. [108]	Milk proteins	ELISA	Thermally treated milk	—	ELISA quantification of milk proteins	2024
84	Freire et al. [109]	Whey protein denaturation	HPLC, ELISA, FTIR, CD	Milk	—	Review of rapid quantification methods	2022
85	Hayes et al. [110]	Skim milk heat stability	ATR-FTIR spectroscopy	Skim milk	—	Real-time measurement of heat stability	2025
86	Zhang et al. [111]	Whey protein changes	CD spectroscopy	Milk	—	Effect of whey protein changes on milk flavor	2024
87	Ren et al. [112]	Milk lipids	Lipidomics (MS)	Milk	—	Progress of MS-based lipidomics in dairy	2023
88	Liu et al. [113]	TAG regioisomers	Lipidomics	Bovine milk	—	Inter-breed and inter-cow variation	2021
89	Rout et al. [114]	Fatty acid distribution	Lipidomics	Human, bovine, buffalo, goat, horse milk	—	Stereospecific distribution of fatty acids	2024

No.	Reference	Detection Target	Analytical Technique	Matrix	LOD/LOQ	Key Performance	Year
90	Wang et al. [115]	Geographical origin	Lipidomics	Sheep milk	—	Lipidomics for geographical origin and breed variability	2025
91	Zhang et al. [116]	Heat-treated milk lipids	Lipidomics	Milk	—	Milk lipids in relation to different heat treatments	2022
92	Rifa'i et al. [117]	Dairy milk microbiota	Metagenomics	Milk	—	Characterization of dairy milk microbiota	2025
93	De Filippis et al. [118]	Dairy microbiome	Shotgun metagenomics	Dairy industry	—	Microbiome mapping in dairy industry	2024
94	Lo et al. [119]	Spoilage bacteria	Metagenomics	Raw milk	—	Heat-stable enzyme genes (aprX, lipA/lipB)	2015
**Regulatory, Foundational and Recent Review References**							
95	Patel et al. [1]	Smart technologies	—	Milk products	—	Leveraging Smart Technologies for safety	2025
96	Ivetić et al. [2]	Sustainable	—	—	—	Innovation in	2025

No.	Reference	Detection Target	Analytical Technique	Matrix	LOD/LOQ	Key Performance	Year
		agriculture				sustainable agriculture	
97	van der Meulen et al. [3]	Food law	—	—	—	EU Food Law Handbook	2020
98	FDA [4]	FD&C Act	—	—	—	US FDA regulatory framework	2018
99	Premanandh et al. [5]	Food fraud	—	—	—	Horse meat scandal—regulatory wake-up	2013
100	Nji et al. [6]	Aflatoxin M1	—	Dairy sector	—	Aflatoxin contamination review	2025
101	Tracy et al. [7]	Dairy regulation	—	China dairy industry	—	Standards recalibration in China	2024
102	Maritano et al. [17]	Food fraud economics	—	High-value food products	—	Anticounterfeiting and fraud mitigation solutions	2024
103	Rasmussen et al. [18]	Dairy cattle diseases	—	Dairy cattle	—	Global losses due to dairy cattle diseases	2024
104	van Asselt et	Food safety	—	European	—	Overview of food safety	2017

No.	Reference	Detection Target	Analytical Technique	Matrix	LOD/LOQ	Key Performance	Year
	al. [10]	hazards		dairy supply chain		hazards	
105	Poonia et al. [12]	Milk adulteration	—	Milk	—	Detection of adulteration in milk review	2017
106	Leduc et al. [14]	Feed restriction	—	Dairy cows	—	Effect of feed restriction on production	2021
107	Casino et al. [15]	Supply chain traceability	—	Dairy sector	—	Blockchain-based traceability	2021
108	Madureira et al. [20]	Bovine whey proteins	—	—	—	Overview of whey protein properties	2007
109	Dekker et al. [21]	Lactose-free products	—	—	—	Lactose-free dairy market and health	2019
110	Cimmino et al. [22]	Milk micronutrients	—	—	—	Role of milk micronutrients in health	2023
111	Claeys et al. [30]	Raw vs. heated milk	—	—	—	Risks and benefits of milk consumption	2013
112	Barbano et al.	True protein	Kjeldahl analysis	Milk	—	Collaborative study on	1991

No.	Reference	Detection Target	Analytical Technique	Matrix	LOD/LOQ	Key Performance	Year
	[31]	content				Kjeldahl method	
113	Winkler et al. [32]	Nitrogen/protein	Dumas method	Milk	—	Nitrogen/protein determination using Dumas	2001
114	Settle et al. [33]	Analytical chemistry	—	—	—	Handbook of Analytical Chemistry Laboratory Practice	2018
115	Archer et al. [34]	Persistent organic pollutants	Automated milk fat extraction	Milk	—	Automated extraction for POPs analysis	2017
116	Crocker et al. [35]	Fat in milk	Gerber vs. Röse–Gottlieb	Milk	—	Comparison of Gerber and Röse–Gottlieb methods	1955
117	Mhapsekar et al. [123]	Milk quality monitoring	IoT, AI	Dairy supply chain	—	Systematic review of IoT and AI in milk quality	2025
118	Xu et al. [124]	Multi-omics in dairy	Multi-omics	Dairy products	—	Research progress in multi-omics analysis of dairy	2026
